# Molecular Survey of Viral Poultry Diseases with an Indirect Public Health Significance in Central Ethiopia

**DOI:** 10.3390/ani11123564

**Published:** 2021-12-15

**Authors:** Behailu Assefa Wayou, Gezahegne Mamo Kassa, Daniela Pasotto, Teshale Sori, Claudia Maria Tucciarone, Mattia Cecchinato

**Affiliations:** 1 Department of Veterinary Microbiology Immunology and Public Health, College of Veterinary Medicine and Agriculture, Addis Ababa University, Bishoftu P.O. Box 34, Ethiopia; newbaye@gmail.com (B.A.W.); gezahegnemamo@gmail.com (G.M.K.); 2 Department of Animal Medicine, Production and Health, University of Padua, 35020 Legnaro, Italy; claudiamaria.tucciarone@unipd.it (C.M.T.); mattia.cecchinato@unipd.it (M.C.); 3 Department of Clinical Studies, College of Veterinary Medicine and Agriculture, Addis Ababa University, Bishoftu P.O. Box 34, Ethiopia; teshalesori2002@yahoo.com

**Keywords:** viral diseases, molecular survey, infectious bronchitis virus, Newcastle disease virus, infectious bursal disease virus, poultry

## Abstract

**Simple Summary:**

Poultry production is increasing, in Ethiopia as well, and poultry is an extremely valuable food resource. This survey investigated the presence of important viral pathogens in poultry (infectious bronchitis virus (IBV), avian metapneumovirus (aMPV), infectious bursal disease virus (IBDV) and Newcastle disease virus (NDV)) using biomolecular assays and sequencing. The results suggested a low circulation of these pathogens, probably owing to vaccination strategies. A routine diagnostic activity should be planned to monitor pathogen circulation and support disease prevention and production levels.

**Abstract:**

The importance of poultry production is globally increasing, in Ethiopia as well, where high-quality protein and contained costs make poultry a valuable food resource. However, this entails some problems linked to rural, backyard and intensively reared flock proximity and pathogen circulation. This study is aimed at monitoring the presence of important viral pathogens in poultry (infectious bronchitis virus (IBV), avian metapneumovirus (aMPV), infectious bursal disease virus (IBDV) and Newcastle disease virus (NDV)) in Ethiopia. Respiratory and cloacal swabs and bursa of Fabricius and kidney imprints on FTA cards were collected in 2021 from 16 farms and tested for IBV, aMPV, NDV and IBDV. One farm was positive for IBDV, resulting in strains similar to those present in vaccines, belonging to genogroup A1a; two farms were positive for IBV but, due to sensitivity limits, only one sample was sequenced, resulting in a 4/91-like strain (GI-13); a layer farm tested positive for NDV with a Lasota-like vaccine strain. These findings suggest a low presence of these pathogens, probably due to the implementation of vaccination strategies, which is also testified by the detection of vaccine strains. A close diagnostic activity should be implemented on a routine basis in order to monitor pathogen circulation, ameliorate biosecurity measures and protect animal health and production levels.

## 1. Introduction

Poultry production is generally hindered by different diseases, and viral agents are among the most frequently occurring pathogens, especially in Ethiopia, where Newcastle disease (ND) and infectious bursal disease (IBD) are some of the major causes of morbidity and mortality [1,2,3,4]. These are high-priority viral poultry diseases in Ethiopia, since intensive poultry farming is growing but it is still flanked by rural and backyard flocks, which greatly differ in their health standards and rearing conditions [3]. To sustain intensive farming, exotic breeds are becoming more and more commonly raised, thus there is a higher host susceptibility due to suboptimal growth and productivity levels. This is considered to complicate the scenario, together with the possible pathogen introduction along with the new breeds [3]. At the same time, large populations of intensively reared chickens are surrounded by small farms and backyard flocks where biosecurity measures are not strict enough, animals of different ages are kept together or birds are not fully vaccinated due to costs, required expertise and the difficulty of purchasing vaccines for private owners [3].

In flocks with no history of vaccination, some authors reported seroprevalence values for NDV ranging between 5–64.1% [5,6], while IBDV seroprevalence can reach 83.1% [7,8,9]. Infectious bronchitis virus (IBV) and avian metapneumovirus (aMPV) were also recently found in Ethiopia at lower occurrence levels [1,2]. 

On the other hand, despite the routine vaccinations being implemented on commercial poultry farms in Ethiopia, NDV outbreaks have been reported and mortality rate remains high [10]. Newcastle disease virus (NDV) is identified as a major killer, largely contributing to economic losses for the poultry sector in Ethiopia, and it is usually the first disease suspected during disease outbreaks [11]. Studies revealed that the majority of the virus strains circulating in village chickens in Ethiopia were virulent strains grouped in the sub-genotype VIf of class II viruses [12].

Infectious bursal disease virus (IBDV) is another common pathogen that usually affects young chickens and weakens their immune system, predisposing the birds to vaccination failure and opportunistic pathogens [13]. The mortality rates in Ethiopian chickens were reported to reach 50% [14]. Low biosecurity standards contribute to the spread of IBDV with risk factors such as visitors from different poultry houses, workers owning rural birds and vendor vehicles aggravating the transmission of the virus [15].

IBDV is an emerging disease in Ethiopia, and it was detected in 2002 for the first time [14]. Its circulation seems to be worsened by the adoption of exotic breeds of chickens, which are considered less resistant than indigenous breeds [16,17]. From the few commercial poultry farms situated in the central part of Ethiopia, in which the disease was first reported, IBDV has widely spread to other parts of the country (Berhanu et al., 2018). Studies have revealed that very virulent IBDV (vvIBDV) strains are circulating in Ethiopia (Shegu et al., 2020), and other work also reported that the Ethiopian IBDVs represented two genetic lineages: the very virulent (vv) IBDVs and variants of the classical attenuated vaccine strain (D78) [18] that is currently adopted, as well as other vaccines based on different strains: B2K, LC75, EXTREM and Winterfield-2512 [17,19].

Despite regular vaccination practices, IBDV is still found in Ethiopia involving both commercial and backyard poultry [20].

Avian infectious bronchitis (IB) is another important disease of poultry that affects the respiratory tract, gut, kidney and urogenital and reproductive systems of chickens [21].

Few studies conducted in different parts of Ethiopia have reported IBV seroprevalence to be high on both commercial and backyard chicken farms. Four serotypes of infectious bronchitis virus were identified from backyard and commercial farms in Ethiopia, namely M41 (GI-1), D-274 (GI-12), 793B (GI-13) and QX (GI-19) [22]. Hutton et al., (2017) also identified a strain belonging to the 793B genotype (GI-13), together with another study reporting the detection of 793B-like (GI-13) strains in distantly spaced backyard flocks, suggesting relevant viral circulation [1].

In these studies [1,2], the authors also detected aMPV subtype B, both in backyard and intensively raised flocks, with respiratory signs. aMPV is a respiratory pathogen whose importance is growing in poultry. Vaccination for aMPV is not commonly adopted in chickens, especially in Ethiopia, where it is currently unavailable [2], so the control of this agent mainly relies on biosecurity. The introduction of aMPV has also been tentatively linked to the importation of birds [1]. Mortality rates are usually low, except for cases of secondary infection that can result in severe forms, in particular with *Escherichia coli* [23].

These infections in poultry deeply affect production but they can also lead to a greater risk for human health, due to the increased susceptibility to both viral and bacterial secondary infection. For example, IBDV does not have any zoonotic potential, but its immunosuppressive nature could favor the replication of pathogens of zoonotic importance, such as *Salmonella* spp., *Campylobacter* spp. and Avian influenza virus [24,25].

Viral diseases are not well studied in most developing countries [2], and the few existing studies in Ethiopia were also largely based on serological tests, rather than on the molecular characterization of the circulating strains [26]. Scarce epidemiological knowledge limits the attempt for control of these diseases and the growth of poultry production.

In order to contribute towards a wider understanding of the epidemiology of the most common viral agents in poultry, the present study was designed to detect the presence of NDV, IBDV, IBV and aMPV and molecularly characterize the circulating strains among poultry farms in the Bishoftu and Mojo towns in Central Ethiopia.

## 2. Materials and Methods

This study was performed in March 2021, and it was centered on Bishoftu and Mojo towns, situated in the East Shewa zone of the Oromia region, Ethiopia. This area was purposely selected based on the large number of poultry farms located here. The sampled farms were further selected based on accessibility and the willingness of the owners to allow the sample collection.

Ten respiratory (pharyngeal and tracheal) and ten cloacal swabs were collected from each visited farm or shed on a farm. Before being pooled, both respiratory (pharyngeal and tracheal) and cloacal swabs were air dried separately for ten minutes and then placed in two different falcon vials. Additionally, FTA card imprints of the bursa of Fabricius and kidneys were collected from dead or moribund chickens humanely euthanized by manual cervical dislocation, on a broiler farm, where mortality was encountered at the time of visit. The FTA card imprints were then pooled based on the farm and shed. Along with the sample collection, different parameters of the farms and study population were recorded, such as number of sheds, total number of birds on the farms, age at sampling, genetic type, vaccination protocol, clinical signs, lesions, applied treatments and morbidity and mortality rates. The anamnestic data were then organized in a comprehensive database.

Samples were briefly stored at +4 °C until the end of the sample collection and then shipped at room temperature to the MAPS Department at Padua University (Italy). Then, laboratory analyses were conducted, and samples were stored at −80 °C until processing, except for FTA cards, which were always kept at room temperature. The pooled swabs were resuspended in 2 mL of PBS and vortexed. FTA card imprints were cut, placed into 1.5 mL tubes, resuspended in 1 mL of PBS and vortexed. A 200 µL aliquot of each resuspended pool was used for nucleic acid extraction with a High Pure Viral Nucleic Acid Kit (Roche, Basel, Switzerland), and then, the extracted samples were stored at −80 °C until further analyses.

Based on the matrix, samples were tested with different molecular assays for different pathogens according to their expected tropism: respiratory swabs were tested for IBV, aMPV and NDV; cloacal swabs were tested for IBV, IBDV and NDV; kidney FTA imprints were tested for IBV; bursa FTA imprints were tested for IBDV.

For aMPV detection and subtyping, a multiplex one-step real time RT-PCR, targeting the G gene [27], was performed using a SuperScript™ III One-Step qRT-PCR System, with a Platinum™ *Taq* DNA Polymerase kit (Invitrogen™, Waltham, MA, USA) on a LightCycler^®^ 96 Instrument (Roche, Basel, Switzerland). Using the same kit and instrument, a preliminary screening for IBV was performed, targeting the UTR region [28], then a one-step RT-PCR, targeting the hypervariable region of the S1 gene, was used for further sequencing and characterization of IBV-positive samples [29]. For NDV detection, a one-step RT-PCR, targeting the F gene, was used [30]. For IBDV, a one-step RT-PCR, targeting the VP2, was used [31].

All RT-PCRs were performed using a SuperScript™ III One-Step RT-PCR System with a Platinum™ *Taq* DNA Polymerase kit (Invitrogen™, Waltham, MA, USA) on an Applied Biosystems 2720 Thermal Cycler (Applied Biosystems, Waltham, MA, USA). Amplicon presence and specificity were examined by agar gel electrophoresis in SYBR™ Safe-stained (Invitrogen™, Waltham, MA, USA) agar gel.

For strain characterization, positive samples for the various pathogens were Sanger sequenced in both directions with the same primer pairs of the RT-PCR assays used for amplification [29,30,31]. Positive samples were prepared for Sanger sequencing and shipped to the sequencing external service of Macrogen Spain (Madrid, Spain). Chromatograms were inspected for quality with FinchTV (Geospiza Inc., Seattle, WA, USA) and assembled in consensus sequences using ChromasPro 2.1.8 (Technelysium Pty Ltd., Helensvale, QLD, Australia). Nucleotide sequences were initially evaluated for specificity using a BLAST [32] search in order to be characterized. For phylogenetic analyses, the database proposed by Valastro et al., (2016) was used for IBV characterization; for IBDV strain characterization, the adopted reference database and classification were those published by Islam et al., (2021); then, for NDV classification, the latest and updated classification approach by Dimitrov et al., (2019) was used [33,34,35]. Sequences were aligned to reference datasets using the MEGA X [36] software for phylogenetic analyses. Phylogenetic trees were reconstructed using the Maximum Likelihood method, and branch support was calculated by performing 1000 bootstrap replicates [36].

## 3. Results

The sampling activity was conducted in March 2021, and a total of 54 pooled samples were collected from 16 farms, located in Bishoftu (10 farms) and Mojo (6 farms). The majority of the animals were layers and were sampled by collecting respiratory and cloacal swabs, while bursa and kidney imprints were collected from six different sheds on a broiler farm. Layers were sampled because of the presence of clinical signs such as torticollis, neck twisting, swollen eyes, eye discharge, dyspnea, salivation, diarrhea, loss of feathers, swollen vent, weakness/listlessness, depression and leg paralysis, and also from apparently healthy farms (five farms). Sampling on the broiler farm was performed because the birds showed hyperemic bursal tissue, a swollen or atrophied bursa and urates in the kidneys at postmortem examination.

When they were applied, treatments ranged from vitamin supplements to antimicrobial drugs (oxytetracycline, sulfadiazine or norfloxacin) to coccidiostats (diclazuril and amprolium hydrochloride). The age of the layers ranged from 3 months to 1 year (mean 9.95 months), whereas broilers were 9 to 23 days old. The genetic types were Bovans Brown and Lohmanns for layers and Cobb 500 for broilers. The population on the layer farms ranged from 150 to 12,000 birds (mean 3528.78), and the mean and median overall mortality rates were 3.25% and 0.33% (range 0–19.23%), respectively, with a higher mortality on smaller farms (rearing less than 300 birds). On some farms, no official records of mortality were kept, and farmers reported the absence of mortality that should actually have been reported as an expected number of deaths, based on the type of birds reared and management levels. The birds were commonly vaccinated against Newcastle disease, infectious bursal disease, Marek’s disease, fowl pox and fowl typhoid, while broilers were vaccinated against Newcastle disease, infectious bursal disease and also infectious bronchitis.

**Figure 1:**
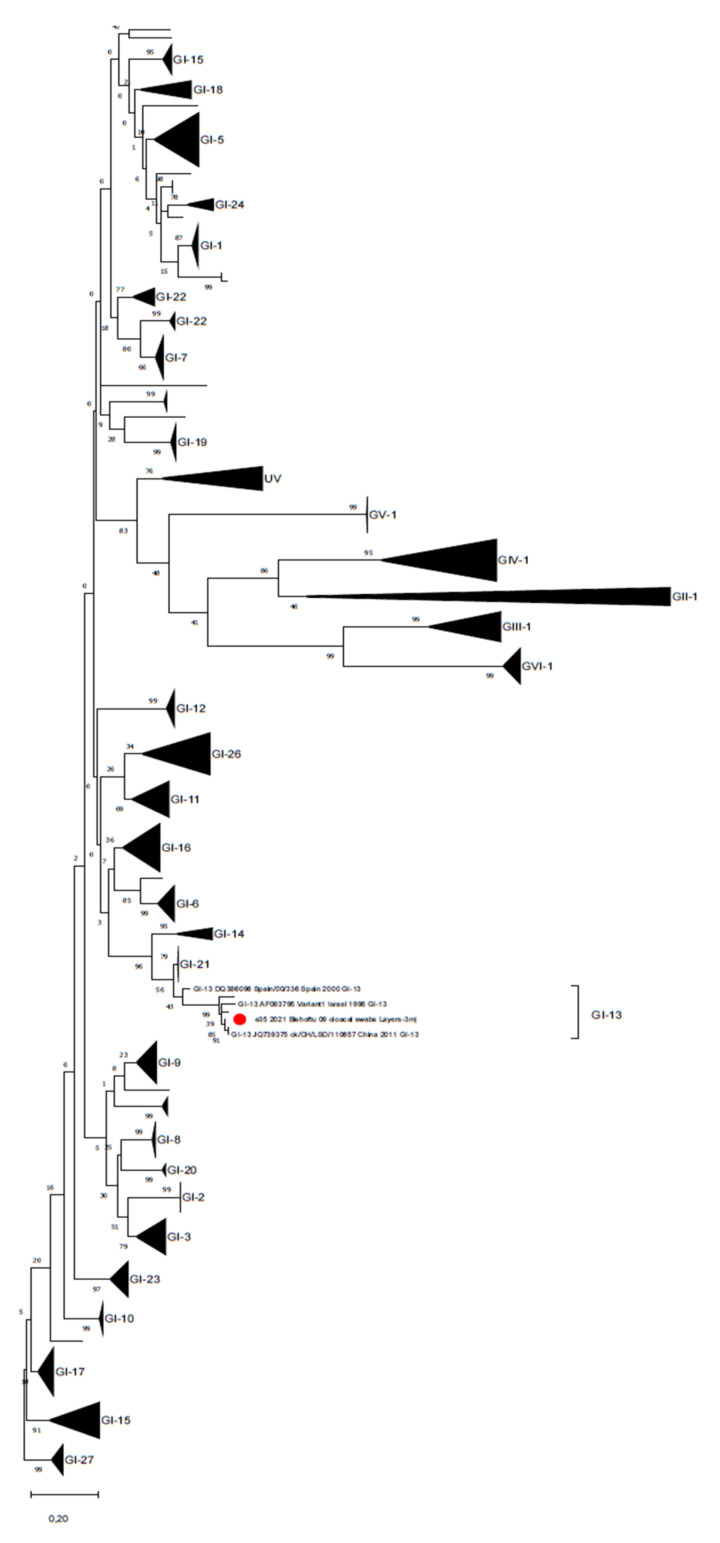
Phylogenetic tree reconstructed for IBV strain characterization with the database proposed by Valastro et al., (2016) [33]. The phylogenetic tree was reconstructed using the Maximum Likelihood method and General Time Reversible model with discrete Gamma distribution. Branch support is shown next to the branches. The Ethiopian strain is marked with a red circle, sequences belonging to the different lineages have been collapsed and single branches represent unique variants.

All samples were negative for aMPV. A total of 2 out of 16 farms (12.5%) (three samples: one cloacal swab pool from a layer farm and two FTA card kidney imprints from the broiler farm) were positive for IBV from real-time RT-PCR screening at high Ct (>38). However, due to sensitivity limits, only one sample was successfully sequenced, resulting in a 4/91-like strain (GI-13) [33] (Figure 1 shows a 99.4% identity with the reference strain MT701511.1).

**Figure 2:**
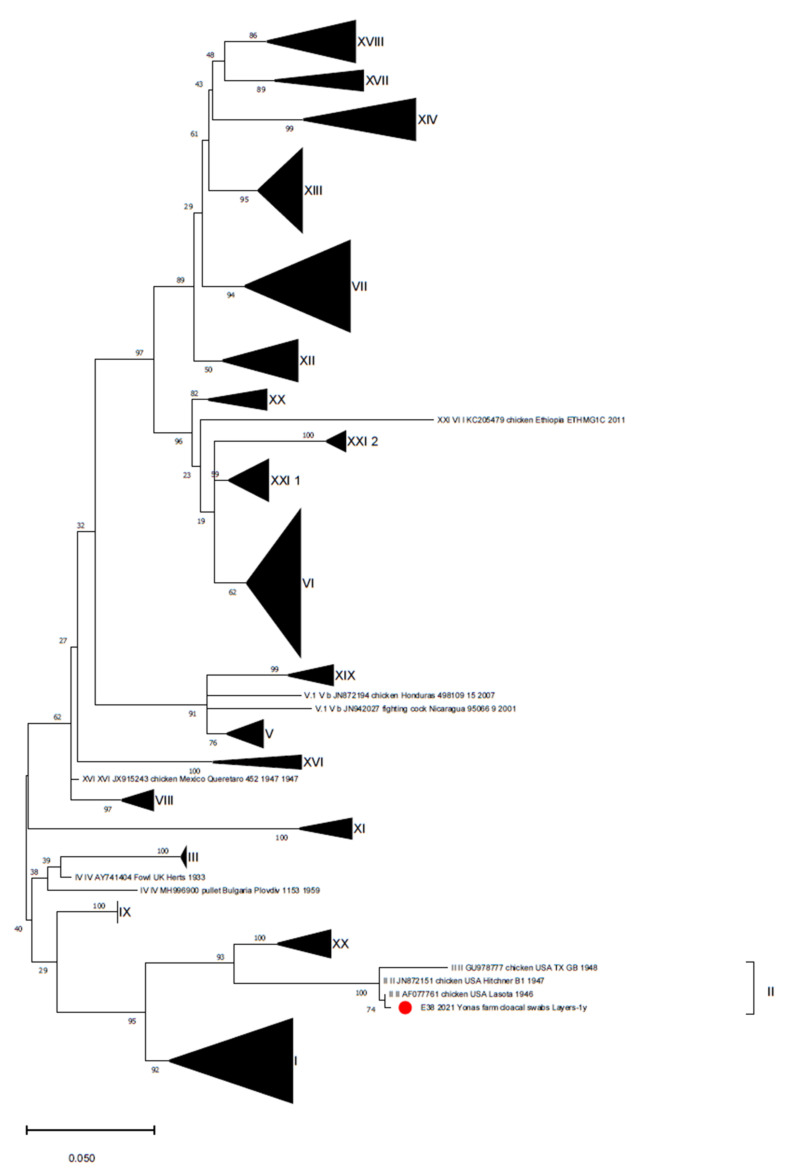
Phylogenetic tree reconstructed for NDV strain characterization with the database proposed by Dimitrov et al., (2019) [35]. The phylogenetic tree was reconstructed using the Maximum Likelihood method and Kimura 2-parameter model with discrete Gamma distribution. Branch support is shown next to the branches. The Ethiopian strain is marked with a red circle.

The sample was collected from a layer farm located in Bishoftu town hosting 3-month-old animals with gastroenteric clinical signs and depression, where birds were reportedly vaccinated with 1/96-based (GI-13) and mass-based (GI-1) vaccines. Only one cloacal swab pool from a Lohmanns layer farm of 1-year-old birds, located in Mojo town, tested positive for NDV (1/15 layer farms, 6.7%), resulting in a vaccine strain close to the Lasota strain (showing a 99.8% identity with strain ID AF077761) belonging to genotype II, based on the updated classification [35] (Figure 2). This vaccine was used in the vaccine protocol implemented on the positive farm, as reported by the farmer.

**Figure 3:**
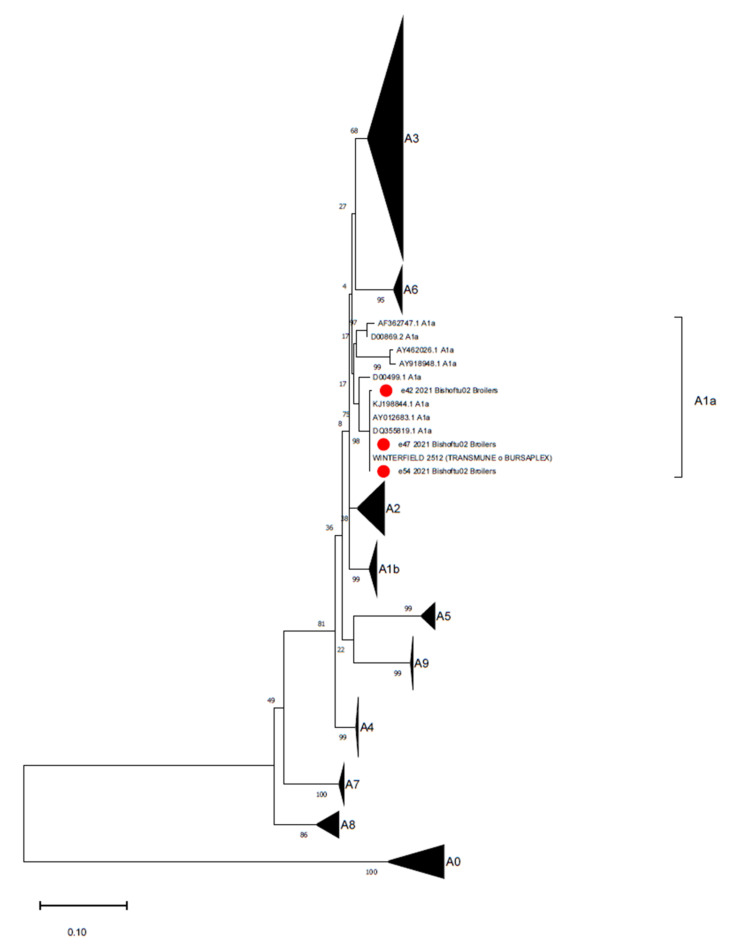
Phylogenetic tree reconstructed for IBDV strain characterization with the database proposed by Islam et al., (2021) [34]. The phylogenetic tree was reconstructed using the Maximum Likelihood method and Kimura 2-parameter model with a discrete Gamma distribution. Branch support is shown next to the branches. The Ethiopian strains are marked with a red circle.

All cloacal swabs from the layer farms tested negative for IBDV, whereas 3 out of 8 bursa imprint pools from the broiler farm (1/16 farms, 6.25%) were positive for IBDV, resulting in highly similar sequences (99.8–100% identity) to the Winterfield-2512 vaccine strain (reference strain MH329181.1), belonging to the classical/virulent genogroup A1a [34] (Figure 3). According to the declared vaccination strategy of the farm, this strain was used for the bird immunizations.

## 4. Discussion

In the present study, detection of the investigated pathogens was limited to four farms only: each farm was positive for a different agent (two farms for IBV, one for NDV and one for IBDV). Unfortunately, it was impossible to further characterize two of the IBV detections from the same farm, while all the characterized strains appeared to be vaccine strains (a 4/91-like vaccine strain, a Lasota-like vaccine strain and a Winterfield-2512-like vaccine strain), indicating either the persistence of the administered vaccine or the spread of vaccine strains from neighboring farms. The positive samples were collected from layer and broiler birds that were reportedly vaccinated against different viral diseases, including those investigated here (IBV, NDV and IBDV), with vaccines based on the detected strains for IBDV and NDV. Regarding IBV detection, the introduction of a vaccine-derived strain from an unknown source (farms implementing a different vaccine protocol, contaminated fomites or personnel) cannot be excluded, since the protocol that was applied on the farm involved different strains.

The persistence of a vaccine strain is a common finding when live vaccines are administered, because they can be shed in feces, followed by re-uptake by the birds and subsequent collection during sampling, thus complicating the diagnostic process. However, the within-flock circulation of live vaccines can also lead to vaccine reactions, when the initial coverage for the birds is only partial [37]. This aspect does not really explain the clinical signs recorded on the positive farms, since different clinical signs (enteric signs, data not shown) to those from typical vaccine reactions were reported on the farm where an IBV vaccine-like strain was detected. Furthermore, on the farm where an NDV vaccine-like strain was detected, no clinical signs were registered, which is desirable. On the broiler farm where IBDV vaccine-like strains were detected, the main recorded lesions were urate deposits in the kidneys.

Even though, in some cases, the reported clinical signs might have been partially suggestive of the investigated pathogens, the actual cause should be ascribed to other problems, most likely of both infectious and managerial origin.

The absence of field strains is surprising, given that the local epidemiology and previous work reported the consistent presence of pathogens such as NDV [5,38], IBV [1,2] and IBDV [39,40]. This finding is also supported by the low mortality rates reported in some cases by the farmers. A certain seasonality, with a higher occurrence of NDV outbreaks during the pre-rainy season, was proposed [41], and the timing of sampling (March) could have influenced the detection rate in the present study. Conversely, all farms declared that they vaccinate their birds, although they did not disclose the complete protocol. The implementation of vaccination surely plays a role in preventing viral circulation, together with possible previous natural infection, and this could have contributed to the acquisition of natural immunity, which was not investigated by serological means in this study.

Vaccination in Ethiopia is often performed on the farm, at the hatchery or at the source, before introducing the animals to the farm, usually starting from one day of age [11]. In fact, Oromia, the region where the study was set, is one of the regions in Ethiopia with the highest accessibility to vaccination and veterinary services, as reported by Aswaf et al., (2021) [42]. 

Vaccination and biosecurity are the key factors in achieving disease control and efficient production, but these measures are often difficult to apply to rural or village farms. However, in this study, small-sized farms (<500 birds) were also free from field strains, suggesting the presence of fair biosecurity levels, prophylactic measures and limited contact with neighboring farms or other potential sources of infection.

## 5. Conclusions

The low circulation of these viruses in this region limits the risks associated to their role as door openers for secondary pathogens, impacting not only poultry production but also public health. Thus, in conclusion, this study displays a reassuring picture of the epidemiological situation in the Oromia region, Ethiopia, and aims to stress the importance of thorough monitoring, information sharing and the implementation of both vaccination strategies and biosecurity measures.
